# Multiparametric Analysis of Cerebral Development in Preterm Infants Using Magnetic Resonance Imaging

**DOI:** 10.3389/fnins.2021.658002

**Published:** 2021-04-13

**Authors:** Marine Dubois, Antoine Legouhy, Isabelle Corouge, Olivier Commowick, Baptiste Morel, Patrick Pladys, Jean-Christophe Ferré, Christian Barillot, Maïa Proisy

**Affiliations:** ^1^Radiology Department, CHU Rennes, Hôpital Sud, Rennes, France; ^2^Inria, CNRS, INSERM, IRISA, Empenn ERL U-1228, Université de Rennes 1, Rennes, France; ^3^Radiology Department, CHU Tours, Hôpital Gatien de Clocheville, Tours, France; ^4^Pediatric Department, CHU Rennes, Hôpital Sud, Rennes, France

**Keywords:** prematurity, magnetic resonance imaging, morphometry, diffusion, cerebral perfusion, arterial spin labeling, radiology

## Abstract

**Objectives:**

The severity of neurocognitive impairment increases with prematurity. However, its mechanisms remain poorly understood. Our aim was firstly to identify multiparametric magnetic resonance imaging (MRI) markers that differ according to the degree of prematurity, and secondly to evaluate the impact of clinical complications on these markers.

**Materials and Methods:**

We prospectively enrolled preterm infants who were divided into two groups according to their degree of prematurity: extremely preterm (<28 weeks’ gestational age) and very preterm (28–32 weeks’ gestational age). They underwent a multiparametric brain MRI scan at term-equivalent age including morphological, diffusion tensor and arterial spin labeling (ASL) perfusion sequences. We quantified overall and regional volumes, diffusion parameters, and cerebral blood flow (CBF). We then compared the parameters for the two groups. We also assessed the effects of clinical data and potential MRI morphological abnormalities on those parameters.

**Results:**

Thirty-four preterm infants were included. Extremely preterm infants (*n* = 13) had significantly higher frontal relative volumes (*p* = 0.04), frontal GM relative volumes (*p* = 0.03), and regional CBF than very preterm infants, but they had lower brainstem and insular relative volumes (respectively *p* = 0.008 and 0.04). Preterm infants with WM lesions on MRI had significantly lower overall GM CBF (13.3 ± 2 ml/100 g/min versus 17.7 ± 2.5, < ml/100 g/min *p* = 0.03).

**Conclusion:**

Magnetic resonance imaging brain scans performed at term-equivalent age in preterm infants provide quantitative imaging parameters that differ with respect to the degree of prematurity, related to brain maturation.

## Introduction

Preterm birth is defined as any live birth occurring before 37 completed weeks’ gestation. The World Health Organization (WHO) subclassifies preterm infants into the following three groups according to their gestational age at birth: extremely preterms (born before 28 weeks’ gestation), very preterms (28–32 weeks’ gestation), and moderate to late preterms (32–37 weeks’ gestation). This frequent problem affected 9.6% of all births worldwide in 2005 (Beck et al., 2010), and the number is increasing every year. The figure rose from 9.8 to 10.8% of all live births between 2000 and 2014 (Chawanpaiboon et al., 2019). Despite constant improvements in those infants’ survival and long-term prognosis, their outcome remains poor. Most preterm children develop motor and cognitive disabilities (Larroque et al., 2008), the severity of which increases with the degree of prematurity. Apart from the usual cerebral complications directly related to preterm birth, such as leukomalacia and intraventricular hemorrhage, prematurity itself can lead to brain development abnormalities with long-term deleterious effects, but the underlying mechanisms remain unknown. Early identification of preterm infants at risk of developing neurodevelopmental disabilities later in life is essential to provide suitable follow-up and more specialized care.

Morphological magnetic resonance imaging (MRI) sequences provide an accurate anatomical analysis of preterm infant brains. At term-equivalent age, extremely preterm children experience an overall brain volume reduction that mainly affects the brainstem and cortical and deep gray matter (GM), in comparison with full-term born infants (Padilla et al., 2015). Regional brain volume variations have also been described in extremely preterm infants, with a volume reduction in the orbitofrontal cortex, precentral and postcentral gyri, temporal cortex, and adjacent deep white matter (WM) areas. Conversely, an increase in relative WM and GM volumes has been observed in vision areas (Padilla et al., 2015). Preterm birth also has an impact on ventricular structure volume and shape (Paquette et al., 2017). In comparison with full-term born infants, preterm infants experience significant ventricular system enlargement that mainly affects the body and frontal horn of the lateral ventricles. Studies that explore the impact of the degree of prematurity on these brain abnormalities remain scarce. Lemola et al. (2017) demonstrated a linear increase in GM and WM volumes with gestational age in preterm infants born between 24 and 32 weeks’ gestation. Furthermore, intellectual quotient results in preterm children linearly increase with gestational age. These results showed a positive correlation between gestational age on the one hand, and cognitive function and brain volume on the other hand. However, the linear relationship between brain volume and gestational age is significant for infants born before 30 weeks’ gestation only. That result suggests that 30 weeks’ gestation is a threshold below which a preterm birth significantly affects long term cognitive performance, instead of the 28 and 32 weeks’ gestation thresholds used in the WHO to classify preterm infants. Nevertheless, brain MRI scans were performed on preterm infants of school age (aged 10) in Lemola’s study. These results warrant confirmation with an early brain MRI scan performed at term-equivalent age, for instance.

Diffusion MRI provides microstructural brain data at a lower scale than morphological sequences through the analysis of water molecular motion in the tissues. By applying several diffusion gradients in different directions, the preferential directions along which the water molecules diffuse can be estimated in each voxel (Le Bihan, 2013). The diffusion tensor imaging (DTI) MRI technique uses the tensor model to characterize the amount and preferred directions of local diffusivity. Several scalar parameters can be derived from the tensor field to quantitatively assess the diffusion properties in each voxel, such as fractional anisotropy (FA), mean diffusivity (MD), axial diffusivity (AD), and radial diffusivity (RD). Diffusion MRI is a well-adapted approach for exploring WM properties and abnormalities (Pecheva et al., 2018; Lebel et al., 2019), and it appears useful for studying WM injury related to prematurity. These WM lesions are common in preterm infants and can lead to subsequent motor or cognitive disabilities. Yet predicting the individual neurological outcome of preterm children is difficult. Kim et al. (2016) showed that FA in preterm infants with WM injuries who go on to develop neurocognitive impairments is significantly lower than FA in preterm infants with WM injuries but with subsequent normal neurological development. This result suggests that DTI could help to predict the neurological outcome of preterm infants with WM injuries.

Brain perfusion is a physiological mechanism whereby oxygen and nutrients are supplied to the brain via microcirculation, and may vary overall or regionally depending on the brain’s metabolic needs. Many methods exist for studying cerebral perfusion in the adult population (Wintermark et al., 2005), and these provide functional data that are complementary to morphological sequences. The arterial spin labeling (ASL) MRI perfusion sequence is a highly suitable technique for imaging brain perfusion in neonates since it is a radiation-free, non-invasive method that uses arterial blood water protons as an endogenous tracer. Furthermore, this technique provides cerebral blood flow (CBF) quantification. Despite these advantages, only a few studies evaluating neonatal brain perfusion through ASL imaging are available due to physiological limitations and technical issues, such as a limited spatial resolution and low signal-to-noise ratio (Proisy et al., 2016). However, this promising tool could reveal subtle abnormalities that cannot be detected with conventional MRI alone. Tortora et al. (2017) showed that overall CBF in GM was higher in healthy preterm infants than in healthy full-term born children, and that overall CBF was significantly lower in preterms with cerebral lesions than in healthy preterms. In addition, Tortora showed that basal ganglia hypoperfusion in preterms is associated with a poor neuromotor prognosis. It has also been reported that overall CBF varies according to the degree of prematurity, with CBF seen as increasing with gestational age (De Vis et al., 2013). These results suggest that ASL perfusion MRI could be used as a non-invasive method to evaluate cerebral maturation in preterm infants, with or without prematurity-related complications.

These previous studies showed that quantitative imaging parameters can be extracted from multiparametric brain MRI images, combining morphological and functional sequences, performed at term-equivalent age in preterm infants. Multiparametric MRI, including morphological, diffusion and perfusion sequences is therefore the most appropriate imaging modality for assessing these brain abnormalities associated with prematurity, and this non-invasive, radiation-free technique is particularly suitable for the pediatric population. We argue that we could identify specific parameters or patterns that vary according to the degree of prematurity or presence of clinical complications, and that those parameters could be used as prognostic markers of subsequent developmental disabilities. The principal aim of this study was to analyze brain volume, scalar diffusion parameters and CBF variations according to the degree of preterm birth. The secondary aim was to evaluate the impact of clinical complications related to preterm birth on brain volume, scalar diffusion parameters and perfusion.

## Materials and Methods

### Population

The data used in this study were part of the Digi-NewB observational cohort^1^ that aims to establish a physiological perinatal database. The Digi-NewB cohort prospectively included newborn infants hospitalized between 2016 and 2019 in the neonatal units of six university hospitals in France (Rennes, Angers, Nantes, Brest, Tours and Poitiers). In this study, we prospectively enrolled a subgroup of the Digi-NewB cohort, consisting of preterm infants admitted to our neonatal referral center from October 2016 to December 2018. Only preterm infants born before 32 weeks’ gestation were included in our study because this preterm subgroup has a higher risk of developing cerebral complications related to preterm birth, such as leukomalacia and ventricular hemorrhage. Thus, based on the Digi-NewB protocol design, a brain MRI scan was systematically performed at term-equivalent age for this sub-group only. We excluded infants with congenital brain abnormalities, genetic, chromosomal, or metabolic disorders, and perinatal hypoxic-ischemic encephalopathy. The infants included were divided into two groups according to their degree of prematurity, following the WHO: extremely preterm who were born before 28 weeks’ gestation and very preterm who were born between 28 and 32 weeks’ gestation. This study was approved by the local ethics committee. All patients’ parents gave written informed consent prior to enrolment.

### Magnetic Resonance Imaging Acquisition

All MRI scans were performed on two clinical devices, both Siemens 1.5T Magnetom Aera scanners (Siemens, Erlangen, Germany). Examinations were performed without sedation during the infants’ natural sleep. The MRI protocol included morphological, diffusion-weighted, and ASL cerebral perfusion sequences. Morphological sequences were 3D T1-weighted images acquired in a sagittal plane. Diffusion-weighted sequences were single-shot EPI (echo planar imaging) spin-echo sequences. The diffusion acquisition consisted of 12 gradient direction images at *b* = 1,000 s/mm^2^ and one b0 image. The two EPI diffusion sequences were acquired with opposite phase-encoding directions: we acquired first a conventional diffusion-weighted sequence combining the 12 *b* = 1,000 s/mm^2^ images and a b0 image with an antero-posterior phase-encoding direction, then we acquired a second b0 image with a posteroanterior phase-encoding direction. Due to protocol variations, two types of morphological and diffusion sequences were performed, and patients underwent either one of these types, or both. The morphological and diffusion-weighted sequence parameters are summarized in Table 1.

**TABLE 1:** Morphological and diffusion weighted sequences parameters.

Parameters	TR (ms)	TI (ms)	TE (ms)	Flip angle (°)	In-plane resolution (mm)	Slice thickness (mm)	Gap (mm)	Field of View (mm)	Turbo factor	Acquisition time (min:s)
**Morphological sequences**										
MPRAGE 1	2,090	1,100	4.92	20	0.51 × 0.51	1	0	512 × 512 × 160	/	5:23
MPRAGE 2	2,040	1,030	4.13	15	0.98 × 0.98	1	0	224 × 224 × 192	2	4:03
**Diffusion-weighted sequences**										
– Diffusion 1	7,100	NA	93	/	1.9 × 1.9	3	0	192 × 192 × 105	192	1:54
– Diffusion 2	2,200	NA	97	/	1.4 × 1.4	4	0	140 × 140 × 104	140	2:27

*Min: minute; s: second; TE: time echo; TI: time inversion; TR: time repetition.*

The perfusion MRI sequence used on both scanners was a 2D pulsed ASL PICORE Q2TIPS sequence (Luh et al., 1999). Imaging parameters were as follows: TR/TE/TI1/TI2 = 3,500/12/700/2,000 ms; in-plane resolution = 4 mm × 4 mm; slice thickness = 8 mm; field of view (FOV) = 256 mm × 256 mm; 9 axial slices with a 2 mm gap. The first volume of the ASL series was used as the M0 reference and 30 label/control image pairs were then acquired. The acquisition time was 3 min and 39 s.

### Data Processing

We adapted our previously developed pipeline (Proisy et al., 2019), initially designed for neonates, to handle our preterm morphological and perfusion data. As for the diffusion data, we implemented a specific in-house pipeline based on the Anima software^2^. These tools were used to quantify cerebral volumes, estimate scalar diffusion parameters and compute average CBF values over a set of regions of interest (ROIs).

**FIGURE 1:**
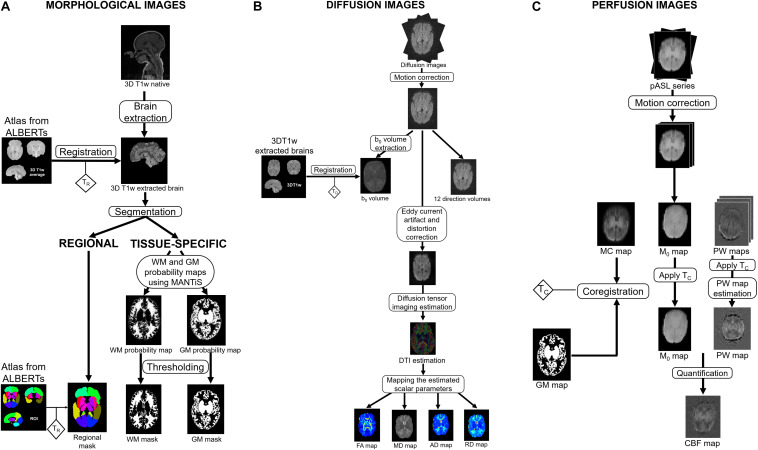
Overview of the processing pipeline for one subject, illustrating the process of morphological **(A)**, diffusion **(B),** and perfusion **(C)** imaging. The transformations T_*R*_, T_*C*_, and T_*D*_ resulting from the different registration steps are represented by diamonds. AD: axial diffusivity, ASL: arterial spin labeling, CBF: cerebral blood flow, FA: fractional anisotropy, GM: gray matter, MC: motion corrected, MD: mean diffusivity, PW: perfusion weighted, RD: radial diffusivity, WM: white matter.

The anatomical processing of our neonate images included brain extraction, tissue segmentation and extraction of ROIs. Our ROIs were derived from a custom anatomical atlas that we built from ALBERTs data (Gousias et al., 2012). The processing of diffusion images led to quantitative scalar diffusion parameters maps for each subject. The processing of perfusion ASL images led to a quantitative CBF map for each subject. Combined with anatomical segmentations, these quantitative maps yielded scalar diffusion parameters and CBF statistics for our ROIs. An overview of the processing pipeline is shown in Figure 1.

#### Morphological Image Processing

A brief review of the morphological images processing is presented below [see Proisy et al. (2019) for a more comprehensive description].

##### Custom anatomical atlas

We first built a custom anatomical atlas from ALBERTs data^3^ which are a set of T1-weighted and T2-weighted brain MRI scans from 20 neonates: 15 preterm children with a mean birth age at 29 weeks’ gestation (26–35 GW) and five full term infants, performed at term-equivalent age. Each of these MRI scans is associated with a manual segmentation of 50 ROIs (Gousias et al., 2012). Using the method developed by Legouhy et al. (2019)^4^ for unbiased atlas creation, our atlas results in an average brain associated with 50 segmented ROIs.

In addition, we created a whole-brain mask from these 50 ROIs for brain extraction purposes.

##### Brain extraction

**FIGURE 2:**
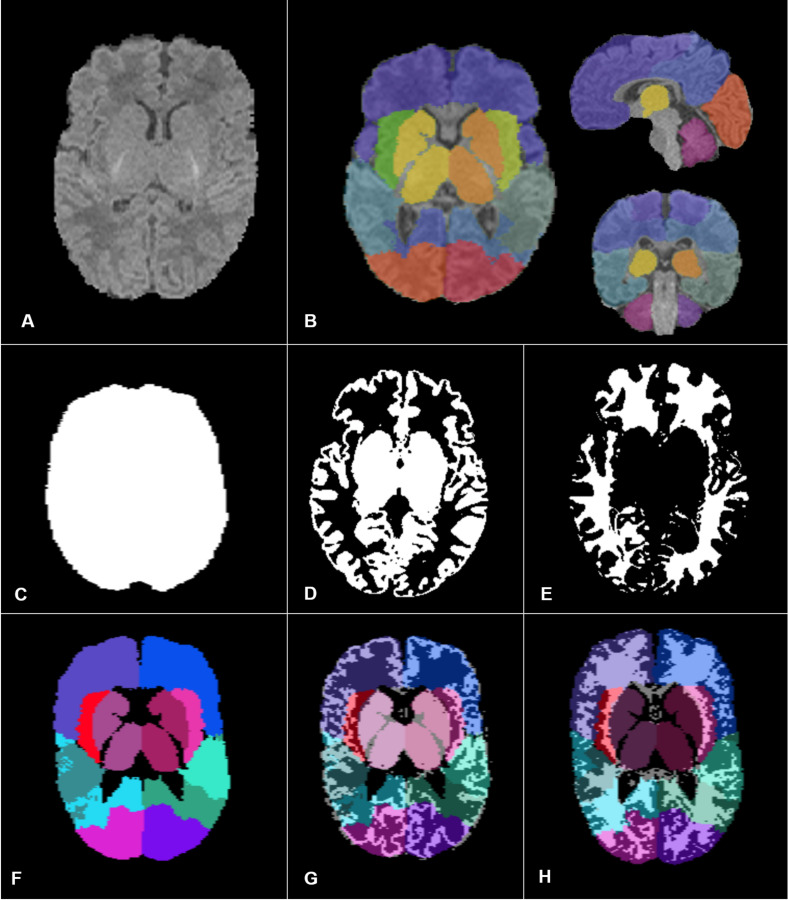
Images and segmentation obtained through our pipeline adapted for preterm infants. We performed brain extraction first **(A)**, then we built, from ALBERTs data, a simplified segmentation of the brain into 14 larger regions **(B)**. To perform MRI quantitative parameters analysis, we calculated our different ROIs: the overall brain **(C)**, the overall GM **(D)**, and WM **(E)**, the eight anatomical regions **(F)**. Panels **(G,H)** correspond to the regional GM ROIs and the regional WM ROIs, respectively.

For each subject, the first step of anatomical processing was brain extraction. The custom atlas built from ALBERTs data was registered onto our subject via an affine transformation followed by a diffeomorphism transformation to perform brain extraction using the whole-brain mask. Henceforth, we will only consider the extracted brain of our subjects (Figure 2A).

##### Regional segmentation

We selected and merged subsets of the 50 ROIs from the segmentation map associated with the custom atlas to form a simplified segmentation of the brain into 14 larger regions: frontal, temporal, occipital, insular and parietal lobes, basal ganglia and thalami for the both right and left sides (Figure 2B). These 14 anatomical regions were first fused in symmetrical pairs to form the seven first ROIs that we analyzed. The eighth ROI was the brainstem volume (region 19 of ALBERTs segmentation). Then, the seven homolateral regions from our 14 larger regions were merged on each side to form two brain hemispheres. The two lateral ventricles’ volumes and corpus callosum volume were also determined using the initial ALBERTs segmentation, to perform the morphological and diffusion data analyses, respectively. For each subject, we used the same registration process as described in our previous study (Proisy et al., 2019) to obtain the corresponding individual regional segmentations for each preterm infant.

##### Tissue segmentation (WM/GM)

The tissue segmentation process used in our previous study was not as accurate as expected for a population of preterm infants. Indeed, in our former method, we used as an *a priori* for tissue segmentation the “Neonatal2 atlas – 44 weeks old, postmenstrual” (Serag et al., 2012), a template tissue probability map adapted for a neonatal population, but the tissue segmentation algorithm was developed for an adult population (Ashburner, 2009). Using a dedicated neonatal tool, we adapted our previous pipeline for tissue segmentation as described below. Tissue segmentation was performed using MANTiS (Morphologically Adaptive Neonatal Tissue Segmentation)^5^, a toolbox from the Statistical Parametric Mapping software (SPM version 8, Wellcome Trust Centre for Neuroimaging, Institute of Neurology, University College London, London, United Kingdom) developed on MATLAB (R2018b version, The MathWorks Inc., Natick, MA, United States). MANTiS uses a template adapted for neonates, and extends the SPM tissue segmentation algorithm that we used in our previous study to a neonate population. The tissue segmentation performances of MANTiS have been tested in preterm infants (born before 30 weeks’ gestation) and Beare et al. (2016) demonstrated that this tool is competitive with other existing methods in this population. The output consisted of eight tissue probability maps (cortical GM, and WM, cerebrospinal fluid, basal ganglia, hippocampus, amygdala, cerebellum, and brainstem). To perform tissue segmentation, MANTiS registered our anatomical images in its specific space. Thus, we registered the eight maps in the anatomical images space with a rigid transformation, and the cortical GM, basal ganglia, hippocampus, amygdala, cerebellum, and brainstem probability maps were merged to obtain the GM probability map. Finally, we thresholded the white and GM probability maps at respectively 0.5 and 0.7 to obtain WM and GM binary maps.

#### Diffusion Data Processing

Diffusion data processing was performed using Anima^6^. Data were prepared to reduce Eddy Current and distortion artifacts which impair scalar diffusion parameter quantification accuracy. To correct distortion artifacts, we used the two acquired EPI sequences with opposite phase-encoding directions: the first and second b0 images were respectively acquired with antero-posterior phase-encoding direction and with posteroanterior phase-encoding direction. The EPI distortion correction was performed using a block matching method that we developed previously (Hédouin et al., 2017). We then performed rigid registration to correct inter-volume motion. For each subject, we next estimated diffusion tensor mapping and extracted the four scalar parameters (FA, MD, AD, and RD). To reduce potential error in the scalar diffusion parameter estimation related to registration, the diffusion data analysis was performed in the diffusion space. Thus, we registered the individual regional masks and WM binary masks onto the corresponding scalar diffusion parameter estimation maps for each subject.

#### Arterial Spin Labeling Perfusion Data Processing

Perfusion data processing was performed using an in-house pipeline adapted to our preterm population, running on MATLAB software (version R2018b) and the SPM toolbox (version 8). Unlike adults, neonates experience huge variations in hematocrit levels, and T1 blood values are directly related to hematocrit levels. Therefore, the preprocessing pipeline was adapted to replace the set T1 blood value usually used for the adult population with a specific T1 value calculated for each subject to improve the accuracy of MRI-derived perfusion measurements (Hales et al., 2016). We also used the GM/WM tissue segmentation maps that we previously reconstructed from MANTiS instead of those used in our previous study (Proisy et al., 2019). As MANTiS was specifically developed for neonates, its tissue maps were more accurate for our preterm population. Aside from these two modifications, we used the same pipeline as in our previous study. The native ASL series were motion corrected, registered and Huber’s M-estimator was used to robustly estimate the PW map. CBF quantification was then performed based on Buxton’s general kinetic model (Buxton et al., 1998) with the following formula:

$$CBF\;=\;6,000\;\times \;\left(\frac{\lambda \;△M\;e^{\left(\left(\frac{{〚\text{TI}〛}_{2}\;+\;{〚idx〛}_{slice\times \;{〚\text{TI}〛}_{slice}}}{{〚\text{T1}〛}_{blood}}\right)\right)}}{\left(2\;\alpha 〚\text{T1}〛1\;{\text{M}}_{0\;blood}\right)}\right)\;[ml\text{/}100g\text{/}min]$$

where 6,000 is the factor for conversion from mL/s to mL/100 g/min, ΔM is the perfusion-weighted map corresponding to the robust mean signal difference between label and control images, and TI_2_ is the time elapsed between the start of labeling and image acquisition. TI_2_ was adjusted to account for the time between acquisition of the different slices of the ROI, with idx_*slice*_ as the slice index (0 for the first slice) and TI_*slice*_ as the time between two successive slices (47 ms). TI_1_ = 700 ms and corresponds to the temporal bolus width. T1_*blood*_ was calculated for each subject using Hales’ formula (Hales et al., 2016) according to the hematocrit level. M_0 blood_ is the longitudinal magnetization of arterial blood at equilibrium and is approximated by the M_0_ map. We used literature-derived values (Alsop et al., 2015) for the following parameters: λ = 0.9 ml/g and is the blood/tissue water partition coefficient and α = 0.98 and is labeling efficiency.

#### Calculation of MRI Statistics Over ROIs

Quantitative parameters were calculated over the ROIs previously described as reported below.

Morphometric analysis was performed at two levels:

Volumes were calculated from the binary segmentations for each ROI separately. We first calculated absolute volumes. Overall volume corresponded to the whole brain volume (Figure 2C). Overall WM and GM volumes were, respectively, the sum of WM and GM voxels in the binary tissue maps (Figures 2D,E). Regional volumes were based on the volumes of the eight anatomical regions defined in section “Regional segmentation” (Figure 2F), the ventricular volumes and the two hemispheric volumes.

Next, we calculated the relative volumes. For each ROI, this involved dividing the region’s absolute volume by the whole brain volume. The intersection of voxels included in each of the ROIs previously described, and the voxels included in the GM and WM binary maps defined the absolute regional volumes for GM (Figure 2G) and WM (Figure 2H). The relative regional volumes for GM and WM were calculated by dividing the regional absolute volumes by, respectively, the overall GM or WM volumes. We did not perform WM volume analysis in the basal ganglia ROI.

Regarding diffusion parameters, the analysis was performed in WM only. For each subject, we calculated the overall WM FA, MD, AD, and RD values, which were the mean of FA, MD, AD, and RD values within the WM across the whole brain. We also calculated the WM regional FA, MD, AD, and RD values that corresponded to the mean of FA, MD, AD, and RD values within the WM of the seven first ROIs defined in section “Regional segmentation” except in the basal ganglia, within the corpus callosum and within the two hemispheres.

Regarding perfusion data, the analysis was performed in GM only. For each subject, we quantified the overall GM CBF, which was the CBF within the GM of the whole brain, and the regional GM CBF, corresponding to the CBF within the GM of the seven first ROIs defined in section “Regional segmentation,” and within both hemispheres.

### Data Analysis

#### Qualitative Analysis

##### Morphological data

Pediatric radiologists with 8–30 years’ experience in pediatric imaging reviewed the morphological sequences to assess potential MRI complications. The infants were classified as having WM lesions if they had one of the following criteria: punctate or extended T1-hyperintense regions in the periventricular WM; cystic WM lesions, appearing as T2-hyperintense and T1-hypointense regions in the periventricular WM, or both. Preterms were classified as having hemorrhage when they had an intraventricular hemorrhage graded as III or IV on the Papile scale (Papile et al., 1978). The quality of anatomical and tissue segmentations was visually checked.

##### Diffusion data

After visual evaluation of the native diffusion-weighted images, we classified the subjects into four groups based on motion artifacts (group 1: no motion artifacts, group 2: inter-volume motion, group 3: intra-volume motion, group 4: intra and inter-volume motion). Group 1 and 2 subjects only were included for data analysis.

##### Perfusion data

Perfusion-weighted maps automatically generated by the MRI scan were rated as having good, moderate, or poor image quality by a pediatric radiologist with 10 years’ experience (MP) and a pediatric radiology resident (MD). Images were graded as good quality when no or only minor artifacts were visible, as moderate quality when moderate artifacts were visible but did not prevent image interpretation, and as poor quality when marked artifacts were visible or images were considered unreadable.

#### Quality Check

As perfusion-weighted maps may show negative values due to the inherent low signal-to-noise ratio and ASL resolution, we calculated the percentage of negative values on the perfusion-weighted maps for GM. For each subject we also automatically extracted the motion range (translation and rotation), signal-to-noise ratio and mean signal.

#### Quantitative Analysis

Statistical analyses were performed, and graphs produced with R-studio (version 1.2.1335©, 2009–2019 RStudio, Inc.), using FactoMineR, factoextra and ggplot2 packages. To assess the quantitative variations in MRI parameters according to the degree of preterm birth, we first built three multiple linear regression models, for morphological, diffusion and ASL perfusion data analyses. Those models allowed us to assess potential linear relations between the quantitative imaging parameters and explanatory variables such as the age at MRI, the sex, the birth weight, and the scanner used to perform the examinations. The models also allowed us to adjust the quantitative imaging parameters to consider potential effects of the variables. Then, we performed a simple linear regression between the adjusted quantitative imaging parameters and the age at birth, and we compared the adjusted quantitative imaging parameters between extremely and very preterm infants. Finally, we compared the adjusted quantitative imaging parameters between preterm infants with and without complications to evaluate the impact of clinical complications related to preterm birth on cerebral volumes, diffusion scalar parameters and perfusion.

##### Demographic data

Continuous variables were expressed as mean ± standard deviations and categorical variables were expressed as counts and percentages. The Gaussian distribution of the included variables was tested using the Shapiro-Wilk test. After assessing distribution normality, continuous variables were compared using Student’s *t*-test, and analysis of variance or the Mann–Whitney *U* test where appropriate. Categorical variables were compared using the chi-squared test. A *p*-value < 0.05 was considered significant for each test performed.

##### Effects of explanatory variables on quantitative imaging parameters

To assess the potential linear effects of explanatory variables on the quantitative parameters extracted from the brain MRI (absolute and relative volumes, diffusion parameters, and CBF), we built multiple linear regression models. Explanatory variables included age on MRI, sex, and the MRI scanner used to assess potential effects on quantitative MRI parameters. We also included, as explanatory variables, all demographics data in which the distribution between extremely and very preterm infants was significantly different in the global population and in the subgroups for diffusion and perfusion analyses, respectively.

• Model used for morphometric analysis:

$$Volume=\beta_{0}+\beta_{1}\times Birthweight+\beta_{2}\times$$

$$\,A\,g\,e\,a\,t\,M\,R\,I+\beta_{3}\times s\,e\,x+\beta_{4}\,s\,c\,a\,n\,n\,e\,r+$$

β5×Maternalhighbloodpressure+β6×

⁢C⁢h⁢o⁢r⁢i⁢o⁢a⁢m⁢n⁢i⁢o⁢n⁢i⁢t⁢i⁢s+β⁢7×P⁢o⁢s⁢t⁢n⁢a⁢t⁢a⁢l⁢s⁢t⁢e⁢r⁢o⁢i⁢d⁢s

+β8×Neonatalinfection+β9×

B⁢r⁢o⁢n⁢c⁢h⁢o⁢p⁢u⁢l⁢m⁢o⁢n⁢a⁢r⁢y⁢d⁢y⁢s⁢p⁢l⁢a⁢s⁢i⁢a+β⁢10

×P⁢a⁢t⁢e⁢n⁢t⁢d⁢u⁢c⁢t⁢u⁢s⁢a⁢r⁢t⁢e⁢r⁢i⁢o⁢s⁢u⁢s+ε

• Model used for diffusion data analysis:

$$\mathrm{Scalar diffusion parameters}=\beta_{0}+\beta_{1}\times B\,i\,r\,t\,h\,w\,e\,i\,g\,h\,t+\beta_{2}\times AgeatMRI+\beta_{3}\times Sex+\beta_{4}\times$$

$$Scanner+\beta_{5}\times Maternalhighbloodpressure+\beta_{6}\times$$

$$M\,a\,t\,e\,r\,n\,o\,f\,e\,t\,a\,l\,i\,n\,f\,e\,c\,t\,i\,o\,n+\beta_{7}\times C\,h\,o\,r\,i\,o\,a\,m\,n\,i\,o\,n\,i\,t\,i\,s+\beta_{8}$$

$$\times P\,o\,s\,t\,n\,a\,t\,a\,l\,s\,t\,e\,r\,o\,i\,d\,s+\beta_{9}\times N\,e\,o\,n\,a\,t\,a\,l\,i\,n\,f\,e\,c\,t\,i\,o\,n+\beta_{10}$$

$$\times Bronchopulmonarydysplasia+\beta_{11}\times$$

Patent⁢ductus⁢arteriosus+ε

• Model used for ASL perfusion data analysis:

$$C\,B\,F=\beta_{0}+\beta_{1}\times B\,i\,r\,t\,h\,w\,e\,i\,g\,h\,t+\beta_{2}\times A\,g\,e\,a\,t\,M\,R\,I+$$

$$\beta_{3}\times S\,e\,x+\beta_{4}\times S\,c\,a\,n\,n\,e\,r+\beta_{5}$$

$$\times Maternalhighbloodpressure+\beta_{6}\times$$

$$M\,a\,t\,e\,r\,n\,o\,f\,e\,t\,a\,l\,i\,n\,f\,e\,c\,t\,i\,o\,n+\beta_{7}\times P\,o\,s\,t\,n\,a\,t\,a\,l\,s\,t\,e\,r\,o\,i\,d\,s$$

$$+\beta_{8}\times N\,e\,o\,n\,a\,t\,a\,l\,i\,n\,f\,e\,c\,t\,i\,o\,n+\beta_{9}$$

$$\times Bronchopulmonarydysplasia+\beta_{10}\times$$

$$\,P\,a\,t\,e\,n\,t\,d\,u\,c\,t\,u\,s\,a\,r\,t\,e\,r\,i\,o\,s\,u\,s+\beta_{11}\times W\,M\,l\,e\,s\,i\,o\,n\,s+\varepsilon$$

For each ROI described in section “Calculation of MRI statistics over ROIs,” we evaluated the effects of the explanatory variables on the quantitative MRI parameters by calculating the β coefficients through multiple linear regression models.

Quantitative parameters extracted from the brain MRI scan were then adjusted in each ROI described in section “Calculation of MRI statistics over ROIs” from their corresponding multiple linear regression models to consider the influence of all the explanatory variables.

##### Effects of the age at birth on quantitative imaging parameters

To assess the potential relation between the age at birth and the quantitative imaging parameters, we built simple linear regression models, with the quantitative imaging parameters as dependent variables and the age at birth as an explanatory variable. For each ROI described in section “Calculation of MRI statistics over ROIs,” we evaluated the effects of the age at birth on the quantitative MRI parameters by calculating the β coefficients through simple linear regression models.

##### Comparison between extremely preterm and very preterm infants

For each ROI described in section “Calculation of MRI statistics over ROIs,” the adjusted value of each quantitative parameter extracted from the brain MRI scan in extremely preterm infants was compared with the one in very preterm infants, using Wilcoxon test. Indeed, owing to the non-normal distribution of most of our data and the small sample sizes, we chose to use non-parametric tests only.

##### Comparison between preterm with and without complications

Six complications were tested: four clinical complications, namely bronchopulmonary dysplasia, patent ductus arteriosus, infection, and necrotizing enterocolitis; and two MRI abnormalities including hemorrhage and WM lesions. For each complication, we built multiple linear regression models based on the ones described in the subsection “Effects of explanatory variables on quantitative imaging parameters,” with the following modifications: the age at birth was added as an explanatory variable and we removed the tested complication from the explanatory variables. Quantitative parameters extracted from the brain MRI scan were adjusted from their corresponding multiple linear regression models to consider the influence of all the explanatory variables. For each complication, we compared the adjusted value of each quantitative parameter extracted from the morphological, diffusion and perfusion sequences for the preterm infants with and without the complication in every ROI described in section “Calculation of MRI statistics over ROIs.” Owing to the non-normal distribution of most of our data and the small sample sizes, we chose to use non-parametric tests only. Thus, we performed Wilcoxon tests.

##### Correction of multiple comparison

Owing to the numerous tests performed, we applied a multiple comparison correction using the Benjamini-Hochberg false discovery rate procedure. Thus, all the initial *p*-values were corrected for multiple hypothesis testing (423 in the morphologic analysis subgroup, 360 in the diffusion analysis subgroup, and 90 in the perfusion analysis subgroup), with a FDR-adjusted *p*-values of < 0.05.

## Results

### Population

We enrolled 34 preterm infants in the study: 13 extremely preterm infants born before 28 weeks’ gestation and 21 very preterm infants born between 28 and 32 weeks’ gestation.

Each included preterm infant underwent morphological sequences. Table 2 summarizes group characteristics for the morphological analysis.

**TABLE 2:** Subject characteristics for morphologic analysis.

	Extremely preterm infants (*n* = 13)	Very preterm infants (*n* = 21)	*p*-value
Age at birth (days)*	**184 ± 7**	**212 ± 8.2**	**< 0.0001**
**Sex**			
Boy	8 (61.5%)	10 (47.6%)	0.501
Girl	5 (38.5%)	11 (52.4%)	
**MRI characteristics**			
Age at MRI (days)*	270 ± 15.1	263 ± 10.7	0.087
MRI scanner			
– Scanner 1	10 (76.9%)	11 (52.4%)	0.15
– Scanner 2	3 (23.1%)	10 (47.6%)	
Morphological sequences:			
– MPRAGE 1	12 (92.3%)	17 (81%)	0.34
– MPRAGE 2	1 (7.7%)	4 (19%)	
**Etiology of prematurity**			
Spontaneous preterm labor	1 (7.7%)	1 (4.8%)	0.73
High blood pressure	**1 (7.7%)**	**11 (52.4%)**	**0.01**
Chorioamnionitis	**5 (38.5%)**	**1 (4.5%)**	**0.02**
Suspicion of infection	11 (84.6%)	10 (47.6%)	0.06
Premature rupture of membranes	6 (46.2%)	4 (19%)	0.14
Metrorrhagia during the second or third trimester of pregnancy	3 (23.1%)	4 (19%)	0.78
**Perinatal**			
Antenatal steroids	12 (97.3%)	21 (100%)	0.38
In-utero growth restriction	1 (7.7%)	3 (14.3%)	0.55
Cesarean delivery	8 (61.5%)	12 (57.1%)	0.80
Birth weight (g)	**921 ± 169**	**1,360 ± 308**	**0.001**
Postnatal steroids	**6 (46.2%)**	**0 (0%)**	**< 0.0001**
**Complications**			
MRI abnormalities	3 (27.3%)	7 (33.3%)	0.52
– Hemorrhage	1 (7.8%)	1 (4.8%)	0.73
– WM lesions	1 (7.8%)	5 (23.8%)	0.21
Clinical abnormalities	**13 (100%)**	**13 (61.9%)**	**0.002**
– Bronchopulmonary dysplasia	**11 (84.6%)**	**7 (33.3%)**	**0.003**
– Patent ductus arteriosus	**13 (100%)**	**8 (38.1%)**	**< 0.0001**
– Infection	**12 (92.3%)**	**9 (42.9%)**	**0.008**
– Necrotizing enterocolitis	2 (15.4%)	1 (4.8%)	0.3

Data are expressed in mean ± sd numbers (percentage). The significant *p*-values are in bold characters. *Age at birth and age at MRI are reported in days of gestation, i.e., from onset of last menstrual period.

Thirty-one of the 34 infants included underwent diffusion-weighted sequences. Of them, eight were excluded by the quality check due to motion artifacts and a further two infants were excluded from data analysis because reliable quantification of the scalar diffusion parameters could not be undertaken due to major distortions remaining after data processing. Finally, statistical analysis was performed on 21 subjects including 10 extremely preterm and 11 very preterm infants. Table 3 summarizes group characteristics for the diffusion analysis.

**TABLE 3:** Subject characteristics for diffusion analysis.

	Extremely preterm infants (*n=10*)	Very preterm infants (*n=11*)	*p*-value
Age at birth (days)*	**185 ± 7**	**213 ± 10**	**< 0.0001**
**Sex**			
Boy	7 (70%)	4 (36.4%)	0.12
Girl	3 (30%)	7 (63.4%)	
**MRI characteristics**			
Age at MRI (days)*	270 ± 16	265 ± 5	0.40
MRI scanner			
– Scanner 1	7 (70%)	5 (45.5%)	0.25
– Scanner 2	3 (30%)	6 (54.5%)	
Diffusion sequences			
– Diffusion 1	5 (50%)	7 (63.4%)	0.53
– Diffusion 2	5 (50%)	4 (36.4%)	
**Etiology of prematurity**			
Spontaneous preterm labor	0 (0%)	1 (9.1%)	0.25
High blood pressure	**1 (10%)**	**8 (72.7%)**	**0.002**
Chorioamnionitis	**4 (40%)**	**0 (0%)**	**0.008**
Suspicion of infection	**9 (90%)**	**3 (27.3%)**	**0.002**
Premature rupture of membranes	4 (40%)	1 (9.1%)	0.09
Metrorrhagia during the second or third trimester of pregnancy	3 (30%)	2 (18.2%)	0.53
**Perinatal**			
Antenatal steroids	9 (90%)	11 (100%)	0.22
In utero growth restriction	0 (0%)	2 (18.2%)	0.1
Cesarean delivery	6 (60%)	9 (81.8%)	0.27
Birth weight (g)	**913 ± 169**	**1,275 ± 337**	**0.006**
Postnatal steroids	**6 (60%)**	**0 (0%)**	**0.001**
**Complications**			
MRI abnormalities	2 (20%)	5 (45.5%)	0.21
– Hemorrhage	1 (10%)	0 (0%)	0.21
– WM lesions	1 (10%)	4 (36.7%)	0.14
Clinical abnormalities	**10 (100%)**	**5 (45.5%)**	**0.002**
– Bronchopulmonary dysplasia	**8 (80%)**	**4 (36.7%)**	**0.039**
– Patent ductus arteriosus	**10 (100%)**	**4 (36.7%)**	**< 0.0001**
– Infection	**10 (100%)**	**4 (36.7%)**	**< 0.0001**
– Necrotizing enterocolitis	2 (20%)	0 (0%)	0.07

Data are expressed in mean ± sd numbers (percentage). The significant p-values are in bold characters.*Age at birth and age at MRI are reported in days of gestation, i.e., from onset of last menstrual period.

Twenty-nine out of the 34 infants included underwent ASL perfusion sequences. Eight infants had poor CBF map image quality. A visual scoring of 3 and a threshold set at 30% of negative values in GM or 5 dB signal-to-noise ratio on perfusion-weighted maps in GM were consistent regarding subject exclusion. Finally, statistical analysis was performed on 21 subjects including 9 extremely preterm infants and 12 very preterm infants. Table 4 summarizes group characteristics for the perfusion analysis. The calculated mean T1_*blood*_ value used for CBF quantification of CBF was significantly higher than the assumed set T1_*blood*_ value at 1.5T (Alsop et al., 2015) (1,754 ± 80 ms versus 1,350 ms, *p* < 0.0001).

**TABLE 4:** Subject characteristics for perfusion analysis.

	Extremely preterm infants (*n=9*)	Very preterm infants (*n=12*)	*p*-value
Age at birth (days)*	**185 ± 8**	**211 ± 7**	**< 0.0001**
**Sex**			
Boy	7 (77.8%)	7 (58.3%)	0.09
Girl	2 (22.2%)	5 (41.7%)	
**MRI characteristics**			
Age at MRI (days)*	**275 ± 14**	**259 ± 14**	**0.016**
MRI scanner			
– Scanner 1	7 (77.8%)	9 (75%)	0.88
– Scanner 2	2 (22.2%)	3 (23%)	
**Etiology of prematurity**			
Spontaneous preterm labor	1 (11.1%)	0 (0%)	0.18
High blood pressure	**0 (0%)**	**7 (58.3%)**	**0.001**
Chorioamnionitis	4 (44.4%)	1 (8.3%)	0.05
Suspicion of infection	**8 (88.9%)**	**5 (41.7%)**	**0.021**
Premature rupture of membranes	5 (55.6%)	3 (25%)	0.15
Metrorrhagia during the second or third trimester of pregnancy	1 (11.1%)	2 (16.7%)	0.72
**Perinatal**			
Antenatal steroids	8 (88.9%)	12 (100%)	0.18
In utero growth restriction	1 (11.1%)	2 (16.7%)	0.72
Cesarean delivery	6 (66.7%)	8 (66.7%)	1
Birth weight (g)	**954 ± 189**	**1,232 ± 299**	**0.018**
Postnatal steroids	**4 (44.4%)**	**0 (0%)**	**0.004**
**Complications**			
MRI abnormalities	1 (11.1%)	5 (41.7%)	0.11
– Hemorrhage	1 (11.1%)	1 (8.3%)	0.83
– WM lesions	**0 (0%)**	**4 (33.3%)**	**0.023**
Clinical abnormalities	**9 (100%)**	**7 (58.3%)**	**0.009**
– Bronchopulmonary dysplasia	**7 (77.8%)**	**4 (33.3%)**	**0.039**
– Patent ductus arteriosus	**9 (100%)**	**4 (33.3%)**	**< 0.0001**
– Infection	**8 (88.9%)**	**4 (33.3%)**	**0.008**
– Necrotizing enterocolitis	1 (11.1%)	1 (8.3%)	0.83

Data are expressed in mean ± sd numbers (percentage). The significant p-values are in bold characters. *Age at birth and age at MRI are reported in days of gestation, i.e., from onset of last menstrual period.

Twelve infants underwent morphological, diffusion and perfusion sequences. Table 5 describes the distribution of morphologic, diffusion and ASL perfusion sequences performed for each included preterm infant.

**TABLE 5:** Distribution of morphologic, diffusion and ASL perfusion sequences performed for each included preterm infant.

Patient	MRI sequences		
	Morphologic	Diffusion	ASL Perfusion
1	MPRAGE 1		PASL
2	MPRAGE 1	Diffusion 1	PASL
3	MPRAGE 1		PASL
4	MPRAGE 1	Diffusion 1	PASL
5	MPRAGE 1	Diffusion 1	PASL
6	MPRAGE 1		PASL
7	MPRAGE 1	Diffusion 1	
8	MPRAGE 1		PASL
9	MPRAGE 1	Diffusion 1	
10	MPRAGE 1		PASL
11	MPRAGE 1	Diffusion 2	
12	MPRAGE 1	Diffusion 1	PASL
13	MPRAGE 1	Diffusion 1	PASL
14	MPRAGE 1	Diffusion 2	PASL
15	MPRAGE 2	Diffusion 1	
16	MPRAGE 1	Diffusion 2	
17	MPRAGE 2	Diffusion 2	
18	MPRAGE 2	Diffusion 2	
19	MPRAGE 1		
20	MPRAGE 1	Diffusion 2	PASL
21	MPRAGE 1		PASL
22	MPRAGE 1	Diffusion 1	
23	MPRAGE 1		PASL
24	MPRAGE 1	Diffusion 1	PASL
25	MPRAGE 1		PASL
26	MPRAGE 1	Diffusion 2	
27	MPRAGE 2	Diffusion 2	PASL
28	MPRAGE 1		
29	MPRAGE 1		PASL
30	MPRAGE 2	Diffusion 1	PASL
31	MPRAGE 1		
32	MPRAGE 1		
33	MPRAGE 1	Diffusion 2	PASL
34	MPRAGE 1	Diffusion 1	PASL

### Effects of Explanatory Variables on Imaging Parameters

#### Morphology

**FIGURE 3:**
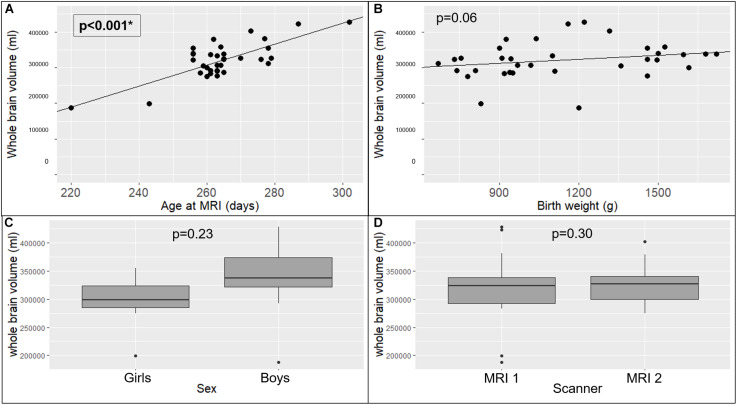
Effects of explanatory variables included in the multiple linear regression model on whole brain volume: age at MRI **(A)**, birth weight **(B)**, sex **(C)**, and MRI scanner used **(D)**. The significant *p*-values are in bold characters within a square frame.

The absolute whole brain volume was significantly positively related with age at MRI (*p* < 0.001) (Figure 3A). The absolute overall GM and WM volumes were also significantly positively related with age at MRI (respectively *p* < 0.001 and 0.001). All regional absolute volumes significantly increased with age at MRI, except for the absolute lateral ventricle volumes. Regarding relative volumes, the relative volume of the right hemisphere significantly increased with the age at MRI (*p* < 0.001) whereas the relative volumes of the basal ganglia, the left hemisphere and the lateral ventricles significantly decreased with the age at MRI (respectively *p* = 0.001, 0.001, and 0.001). The age at MRI was also significantly negatively related to the relative volumes of GM in the basal ganglia (*p* = 0.006), and significantly positively related to the relative volume of GM in the right hemisphere (*p* = 0.009) and to the relative volumes of WM in the temporal areas (*p* < 0.001).

The birth weight had no significant impact on the absolute whole brain volume (*p* = 0.065) (Figure 3B), but the absolute volumes of the brainstem, the basal ganglia, and in the temporal, cerebellar and insular lobes significantly increased with the birth weight (respectively *p* = 0.01, 0.01, 0.03, 0.04, and 0.04). None of the relative volumes were significantly related to the birth weight.

The absolute whole brain volume was not significantly different in preterm boys than in preterm girls (340.98 ± 49.7 ml versus 298.32 ± 25.9 ml, *p* = 0.23) (Figure 3C). The absolute insular volume was the only volume that was significantly related to the sex (*p* = 0.04). Regarding the other absolute volumes and the relative volumes, we did not find any other significant relation between sex and any of the tested ROIs. The scanner type had no significant effect on the absolute whole brain volume (308.1 ± 53.7 ml versus 325.7 ± 27.8 ml, *p* = 0.30) (Figure 3D), nor on the absolute GM or WM volumes (respectively *p* = 0.28 and 0.44). Regarding regional absolute and relative volumes, we did not find any significant relation between the scanner type and any of the tested ROIs.

Chorioamnionitis significantly affected several regional absolute volumes, namely the absolute volume of the brainstem (*p* = 0.01), the basal ganglia (*p* = 0.01), and the insula (*p* = 0.04) that significantly decreased when chorioamnionitis was present. It had no significant effects on the relative volumes.

A neonatal infection, maternal high blood pressure and patent ductus arteriosus had no significant effect on the absolute whole brain volume (respectively *p* = 0.5, 0.65, and 0.75), or on any of the tested ROIs.

#### Diffusion

The age at MRI had a significant positive relation with the FA in WM of the whole brain (*p* = 0.009) and the corpus callosum (*p* = 0.01), and in the WM of all the tested ROIs except for the temporal, cerebellar and occipital areas. The age at MRI had a significant negative relation with the MD, AD and RD of the WM within the cerebellar and occipital areas.

None of the other explanatory variables (sex, the scanner used, birth weight, maternofetal infection, chorioamnionitis, patent ductus arteriosus, bronchopulmonary dysplasia, or postnatal steroids) had a significant effect on scalar diffusion parameters.

#### Arterial Spin Labeling Perfusion

Regarding the other explanatory variables, we did not find any significant relation between the overall CBF within the GM and the age at MRI (*p* = 0.47), sex (*p* = 0.29), the scanner used (*p* = 0.74), or the birth weight (*p* = 0.57), neither with the other explanatory variables. These variables had no significant effect on any regional CBF in the GM either.

### Correlation Between the Gestational Age at Birth and Imaging Parameters

#### Morphometry

The gestational age at birth had no significant effect on the absolute whole brain volume (*p* = 0.75), or on the absolute overall GM or WM volumes (respectively *p* = 0.86 and 0.85). Regarding the relative volumes, the insular relative volume significantly increased with the age at birth (*p* = 0.002). None of the other absolute or relative volumes were significantly related to the age at birth.

#### Diffusion

The gestational age at birth was not significantly related to the FA, the DM, AD, or RD values in the WM (respectively *p* = 0.98, 0.98, 0.98, and 0.98). None of the regional scalar diffusion parameters were significantly related to the age at birth.

#### Arterial Spin Labeling Perfusion

Gestational age at birth had no significant effect on the CBF across the GM of the brain (*p* = 0.15). However, the age at birth was significantly related to several regional CBF across the GM, namely the occipital and left hemispheric CBF across the GM that decreased with the age at birth (respectively *p* = 0.003 and 0.03).

### Comparison Between Extremely Preterm and Very Preterm Infants

#### Morphometry

Absolute whole brain volumes were not significantly different between the extremely preterm infants when compared to very preterm infants (332.26 ± 55.5 ml versus 313.88 ± 37.3 ml, *p* = 0.65). There was no significant difference regarding absolute GM (*p* = 1), WM (*p* = 0.97), or regional volumes. The absolute volume of lateral ventricles was not significantly different between the two groups either (7.1 ± 0.5 ml versus 6.8 ± 0.8 ml, *p* = 0.5).

**FIGURE 4:**
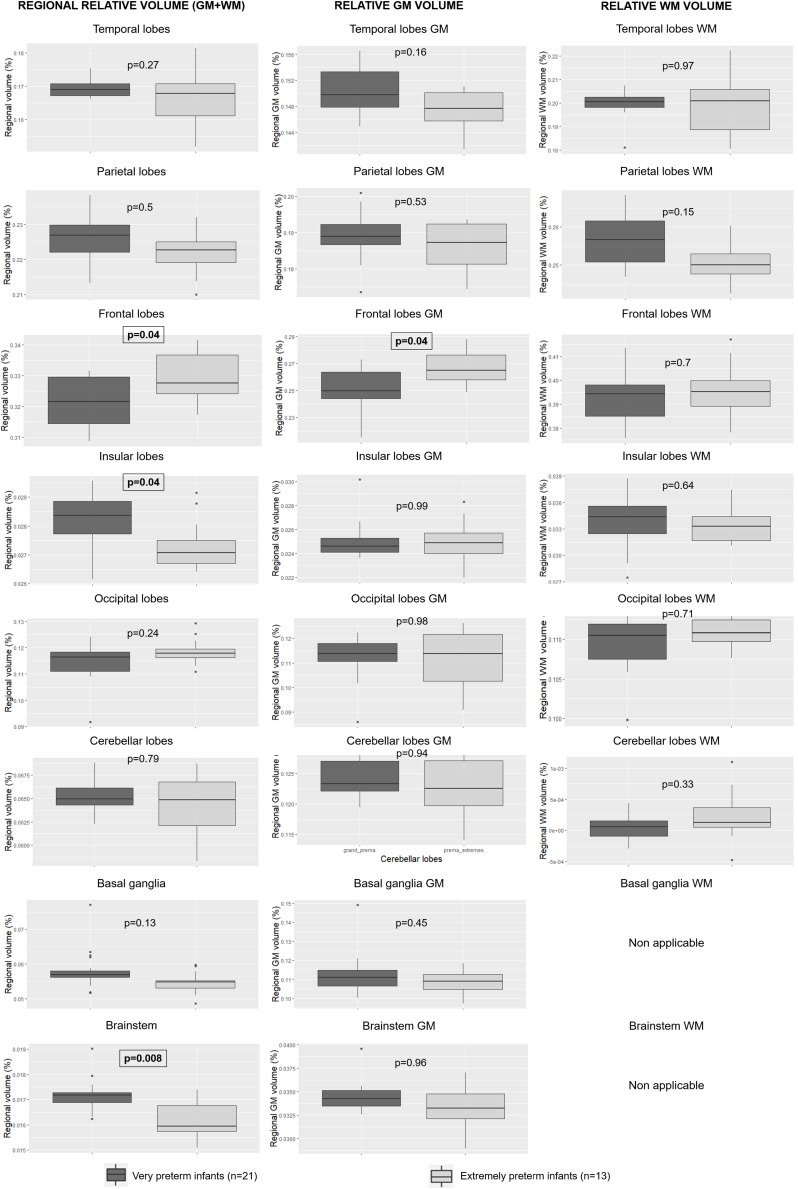
Relative regional, GM and WM volumes according to degree of prematurity. GM: gray matter; WM: white matter. The significant *p*-values are in bold characters within a square frame.

However, the analysis of relative regional volumes showed several significant differences between the two groups (Figure 4): extremely preterm infants had significant higher frontal overall and frontal GM relative volumes (respectively *p* = 0.04 and 0.04). Very preterm infants had significant higher relative volumes within the brainstem (*p* = 0.008) and the insula (*p* = 0.04).

#### Diffusion

**FIGURE 5:**
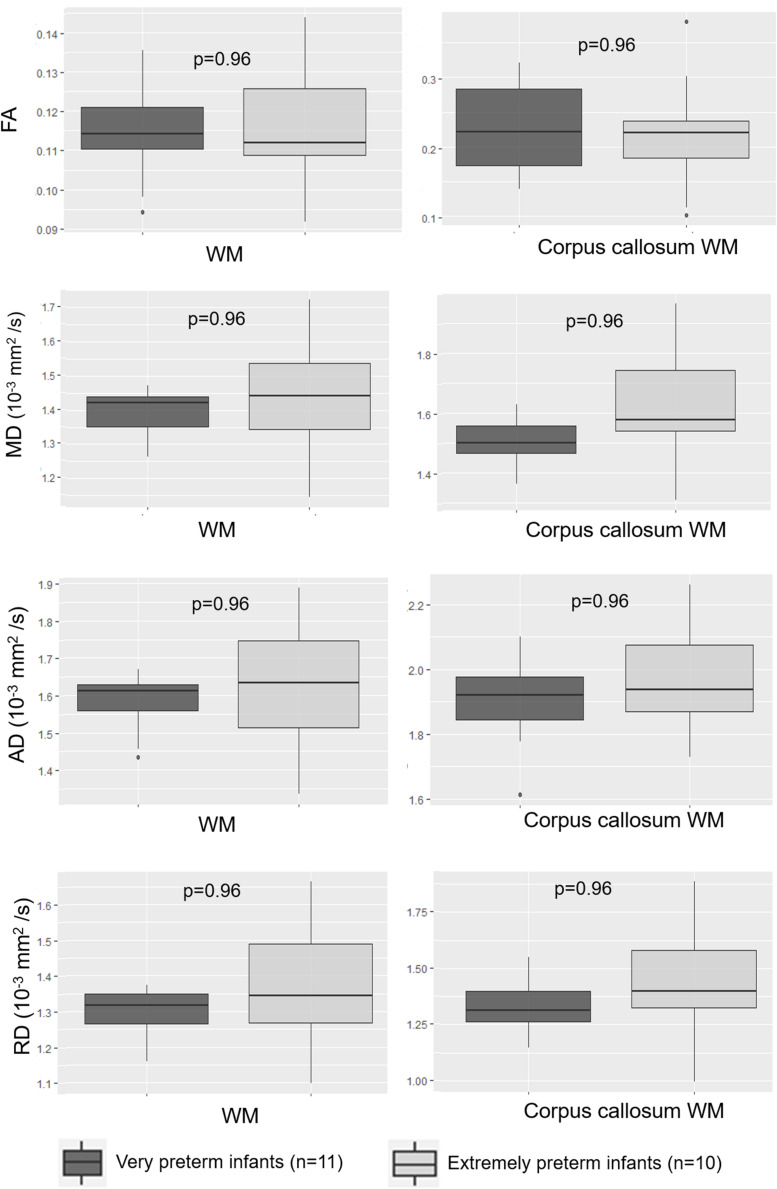
Scalar diffusion parameters for the whole WM and corpus callosum WM according to degree of prematurity. AD: axial diffusivity; FA: fractional anisotropy; MD: mean diffusivity; RD: radial diffusivity; WM: white matter.

There was no significant difference in FA, MD, AD, or RD values within the WM whole brain (respectively *p* = 0.96, 0.96, 0.96, and 0.96), or corpus callosum (respectively *p* = 0.96, 0.96, 0.96, and 0.96) between extremely preterm and very preterm infants (Figure 5).

#### Arterial Spin Labeling Perfusion

**FIGURE 6:**
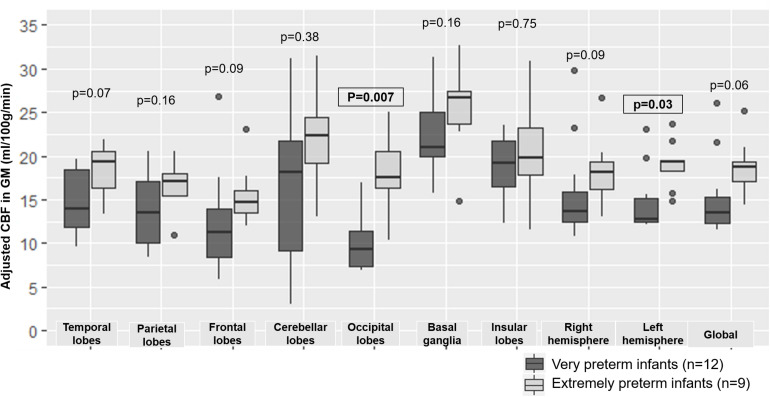
Boxplot representing regional and overall CBF according to degree of prematurity. CBF: cerebral blood flow, GM: gray matter. The significant *p*-values are in bold characters within a square frame.

The CBF within the GM of whole brain was not significantly different in extremely preterm infants when compared to very preterm infants (18.6 ± 3.2 versus 15.1 ± 4.4 ml/100 g/min, *p* = 0.06). However, significant differences were observed in regional GM CBF according to the degree of prematurity in the occipital lobes (*p* = 0.007) and left hemisphere (*p* = 0.03) (Figure 6).

### Comparison Between Preterms With and Without Complication

#### Morphometry

Regarding clinical complications, the presence of bronchopulmonary dysplasia was related to significant variations of relative volumes, with a significant decrease of the insular relative volume (*p* = 0.012), the temporal relative volume (*p* = 0.003) and the GM temporal relative volume (*p* = 0.002) in preterm with bronchopulmonary dysplasia. The presence of necrotizing enterocolitis significantly affected regional volumes, mainly affecting the absolute volumes. Preterm infants with necrotizing enterocolitis had significantly lower absolute volumes of the brainstem (*p* = 0.024), the basal ganglia (*p* = 0.02), the insula (*p* = 0.04), the cerebellar (*p* = 0.04), and temporal lobes (*p* = 0.03), and a lower relative temporal volume (*p* = 0.04) when compared to preterm infants without necrotizing enterocolitis.

The presence of neonatal infection or patent ductus arteriosus did not have a significant impact on the absolute or relative volumes in any of the tested ROIs.

Regarding macroscopic imaging findings, none of the absolute or relative regional volumes were significantly affected by the presence of WM lesions or brain hemorrhage.

#### Diffusion

The presence of necrotizing enterocolitis had no significant effect on the FA within the WM whole brain or within the WM of the anatomical regions. However, the MD and RD within the WM whole brain were significantly higher in preterm infants with necrotizing enterocolitis than in preterm infants without necrotizing enterocolitis (respectively *p* = 0.036 and 0.036). The presence of necrotizing enterocolitis was also related to a significant increase of the MD and RD in every WM region except for cerebellar and occipital areas, and to a significant increase of AD in parietal and frontal lobes, in the corpus callosum and in both hemispheres.

None of the other clinical complications or MRI abnormalities were related to significant variations of the diffusion scalar parameters values within the WM whole brain or in the regional tested ROIs.

#### Arterial Spin Labeling Perfusion

**FIGURE 7:**
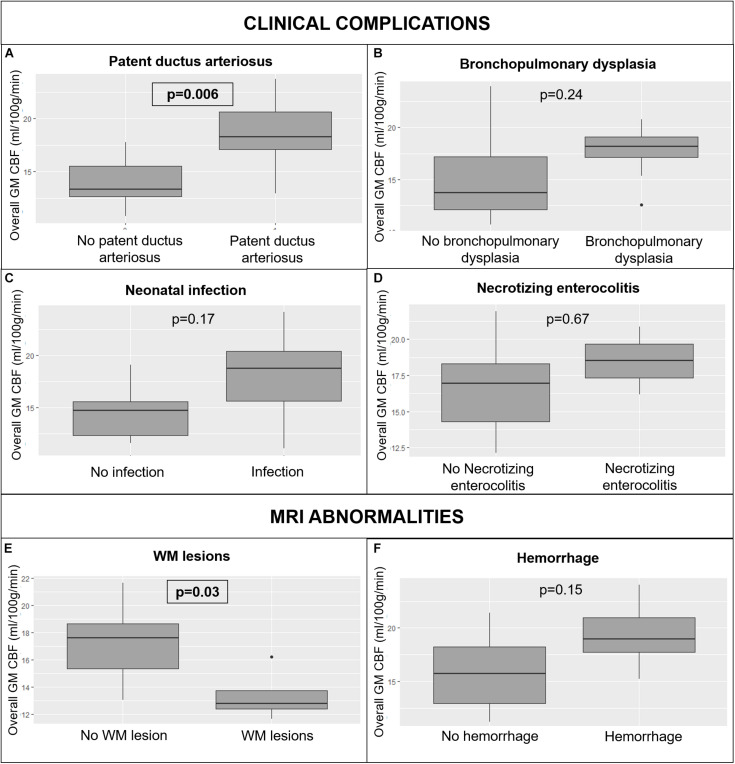
Variations in whole brain GM CBF in preterm infants according to clinical complications: patent ductus arteriosus **(A)**, bronchopulmonary dysplasia **(B)**, neonatal infection **(C)**, and necrotizing enterocolitis **(D)**; and MRI abnormalities: WM lesions **(E)** and hemorrhage **(F)**. CBF: cerebral blood flow, MRI: magnetic resonance imaging, WM: white matter. The significant *p*-values are in bold characters within a square frame.

Regarding clinical complications, a patent ductus arteriosus was related to significant differences in overall GM CBF (18.2 ± 3.2 ml/100 g/min versus 13.9 ± 2.2 ml/100 g/min; *p* = 0.006) (Figure 7A) and regional GM CBF in the frontal (15.6 ± 3.4 ml/100 g/min versus 10.1 ± 3.3 ml/100 g/min, *p* = 0.006) and occipital lobes (16.2 ± 3.6 ml/100 g/min versus 8.9 ± 2.1 ml/100 g/min, *p* < 0.001) and left (18.3 ± 2.7 ml/100 g/min versus 13.7 ± 2 ml/100 g/min, *p* = 0.006) and right hemispheres (18.3 ± 4 ml/100 g/min versus 14.2 ± 2.6 ml/100 g/min, *p* = 0.04) when compared to preterms without patent ductus arteriosus. The presence of bronchopulmonary dysplasia (Figure 7B), an infection (Figure 7C) or necrotizing enterocolitis (Figure 7D) was not associated to a significant CBF variation within the GM whole brain or in the regional tested ROIs.

Regarding the MRI abnormalities, preterm infants with WM lesions on MRI had significantly different overall GM CBF (13.3 ± 2 ml/100 g/min versus 17.7 ± 2.5, <ml/100 g/min *p* = 0.03) (Figure 7E), and significantly different CBF in the occipital lobe, and the left hemisphere GM CBF when compared with preterm infants without WM abnormalities (respectively *p* = 0.03 and 0.03). Hemorrhage had no effect on overall GM cerebral perfusion (17.9 ± 3.7 ml/100 g/min versus 16.2 ± 2.5 ml/100 g/min, *p* = 0.15) (Figure 7F), neither on the regional GM perfusion.

## Discussion

Brain segmentation and extraction methods commonly used in adult patients are far less effective in neonates, mainly due to differences in brain tissue contrast. WM and GM contrast is inverted compared to adult brains, which can be explained by the incomplete myelination process. Therefore, we adapted the pipeline that we previously developed for neonates (Proisy et al., 2019), to fit our preterm population using a tissue segmentation tool dedicated to neonates. We also changed the blood T1-relaxation time used to calculate CBF values. The T1_*blood*_ value depends on the hematocrit rate, which is highly variable in neonates (Jopling et al., 2009), unlike in adult populations. The longitudinal relaxation time of blood used for CBF quantification is generally assumed to be 1,350 ms in neonates (Alsop et al., 2015), but this assumption does not account for the high variability in this population and can lead to a 10% error in CBF quantification (De Vis et al., 2014). In our study, we calculated a specific T1_*blood*_ value for each subject according to their own hematocrit rate using Hales’ technique (Hales et al., 2016). The resulting mean T1_*blood*_ value was significantly higher than the assumed set T1_*blood*_ value at 1.5T (Alsop et al., 2015) (1,754 ± 80 ms versus 1,350 ms, *p* < 0.0001). This result was consistent with the longitudinal relaxation time of blood measured by De Vis et al. (2014) in neonates on a 3T MRI scanner, which was higher than the assumed set T1_*blood*_ value at 3T (1,890 ms versus 1,650 ms) (Alsop et al., 2015).

In our study, multiparametric brain MRI performed at term-equivalent age provides quantitative parameters that are significantly different between extremely preterm and very preterm infants.

ASL perfusion was the most discriminating MRI sequence for separating preterm infants according to degree of prematurity. Our whole brain mean CBF value (18.2 ± 1.6 ml/100 g/min) was consistent with literature reports (19.2 ± 4.3 ml/100 g/min) (Bouyssi-Kobar et al., 2018). Studies evaluating CBF quantification with respect to the degree of prematurity are scarce and results are contradictory. De Vis et al. (2013) showed a positive correlation between age at birth and CBF from 31 weeks’ gestation to term-equivalent age, whereas Tortora et al. (2017) reported that mean CBF in preterm infants was significantly higher when compared to full-term born infants. Our results showed that the overall CBF in the GM and all the regional GM CBF values tend to be higher in extremely preterm than very preterm infants, although the differences were only significant in occipital and left hemispheric GM after multiple comparison correction. Therefore, our results were consistent with the latest studies including Tortora’s one. In addition, given the boxplot in Figure 6, it seems that preterm infants present regional CBF disparities with higher brain perfusion in insular lobes, cerebellar lobes and basal ganglia when compared with other brain regions. These disparities were also described using nuclear medicine methods: Tokumaru et al. (1999) reported predominant uptake in the basal ganglia, brainstem and cerebellum with relatively less cortical activity in the perinatal period. The main theory explaining the regional differences is that neural activity, and therefore cerebral perfusion, are higher in those areas because they are involved in primary responses to sensory stimuli. Maturation of the basal ganglia and cerebellum occurs earlier in preterm infants than full-term infants because their extrauterine life is longer at term-equivalent age. Lastly, our results showed that extremely preterm infants had significantly higher occipital CBF than very preterm infants, which corresponds to the visual cortex. This result was also reported by Smyser et al. (2016) and confirmed the theory that maturation of sensory areas occurs earlier than other functional brain areas.

Regarding morphometric parameters, we found that age at MRI was significantly correlated with the absolute overall volume and the age at MRI and birth weight were significantly correlated with the absolute overall and most regional volumes. The age at MRI was also significantly related to numerous regional, GM and WM relative volumes. Those results show that several variables may influence cerebral volumes, as previously reported by Gousias et al. (2012). Thus, the multiple linear regression models used to adjust brain volumes in this study are justified to take the explanatory variables into account. Interestingly, many absolute regional volumes were significantly positively related to birth weight, but none of the relative volumes were. These findings are consistent with the study of Tolsa et al. (2004), which showed that preterm infants with in-utero growth restriction had significantly lower global absolute brain volumes than control infants. It also suggests that birth weight influences all the absolute volumes in consistent proportions and has no impact on the ratio of each brain lobe within the whole brain. In addition, none of the adjusted absolute or relative volumes, except one, were significantly different between boys and girls. This finding is contradictory with the study of Gilmore et al. (2007), that showed that at birth, boys have larger whole GM and WM volumes than girls. In our study, cerebral volumes were adjusted to consider the potential influence of other variables, such as birth weight. It suggests that sex has no direct effect on brain volumes, but could be related to variations in birth weights.

In this study, the age at birth was found to have no significant relation with the absolute or the relative cerebral volumes except the insular relative volume. Several reasons could explain this lack of significance. The subtentorial brain parenchyma, brainstem and cerebellum grow linearly during the third trimester of pregnancy (Bouyssi-Kobar et al., 2016), but nonlinear growth may be possible in smaller brain areas, and our multiple linear regression model may be too simplistic. However, it was the most effective tool available for taking all the explanatory variables. Another hypothesis is that the number of included infants may be insufficient to highlight potential significant differences. However, we found several significant relative volume variations between extremely and very preterm infants. This result is partially consistent with the study of Lemola et al. (2017), who found that the impact of prematurity on cerebral volumes is less severe in infants born after a certain gestational age threshold. But unlike Lemola et al. who proposed to use the 30 weeks’ gestational age at birth threshold, we showed that the 28-week gestation threshold used by the WHO is a relevant age at birth threshold to separate preterm infants into different groups according to cerebral volumetric variations. Nevertheless, it would be interesting to test which age at birth threshold is the most relevant to identify a subgroup of preterms more vulnerable to morphological variations related to prematurity in a dedicated study.

None of the absolute volumes were significantly different between extremely and very preterm infants. However, we found more significant differences between those two groups when we analyzed relative regional volumes rather than absolute regional volumes. It suggests that, unlike the influence of birth weight, the degree of prematurity does not influence absolute volumes in consistent proportions. Therefore, it has an impact on the ratio of each brain lobe within the whole brain. Thus, the relative regional volume analysis is more relevant to study the effects of the degree of prematurity on the morphological brain maturation process.

Furthermore, the relative volume variations that we highlighted in preterm infants seem to be related to the brain maturation process. Extremely preterm infants had significantly higher relative frontal when compared to very preterm infants. The primary motor area is in the frontal lobe. This difference could be explained by earlier maturation in this motor area in extremely preterm infants than in very preterm infants, related to a longer extrauterine life at term-equivalent age and therefore longer exposure to sensory and motor stimuli (Alexander et al., 2019). Inversely, the insular relative volumes were significantly lower in extremely preterm infants when compared to very preterm infants. Those differences could be explained by the functional role of the insular lobes which oversee superior functions such as memory, language, emotions, and which develop later than the primary sensory and motor functions.

In our study, diffusion parameters were not significantly correlated with the age at birth and did not differ between extremely and very preterm infants. Rather, Shim et al. reported a positive correlation between the age at birth and overall FA, a negative correlation between the age at birth and MD, and observed that FA was significantly lower in preterm infants when compared with full-term born infants (Shim et al., 2012). Furthermore, in our study, the age at MRI was significantly positively related to the FA in the WM of the whole brain and of numerous regions. Additionally, it was significantly negatively related to the MD, the AD and the RD in the WM of the cerebellar and occipital lobes. We may hypothesize that the scalar diffusion parameters are related to the gestational age of infants at term-equivalent age but the gestational age at birth, which reflects the proportion of extrauterine life at term-equivalent age does not impact the diffusion parameters. These discordant results in the literature may also be explained by other factors. To analyze diffusion data, we used anatomical region-based segmentation. Functional regional segmentation based on WM fiber bundles might have been more appropriate, but we opted to use the same anatomical segmentation to analyze diffusion, ASL perfusion and morphometric data to ensure standardization of results. Also, the age at birth threshold chosen at 28 weeks’ gestation may not be optimal for identifying diffusion parameter variations. Indeed, the critical age at birth below which the developmental trajectory of gray and WM structures in the brain is altered is region- and diffusion parameter specific (Wu et al., 2017). Lastly, the number of included infants may be insufficient for identifying potential significant differences.

Several clinical complications had an impact on morphologic and perfusion metrics. Preterm infants with bronchopulmonary dysplasia had morphometric-related brain changes, and preterm infants with patent ductus arteriosus had perfusion-related brain changes when compared with preterm infants without those clinical complications. However, these results are biased and difficult to interpret because extremely preterm infants had significantly more clinical complications than very preterm infants. In our study, preterm infants with patent ductus arteriosus had significantly higher overall and regional CBF than preterm infants without any complications, whereas the opposite was reported in the literature (Bouyssi-Kobar et al., 2018). Furthermore, several regional brain volume variations in preterm infants with bronchopulmonary dysplasia were the same as those in extremely preterm infants. Thus, CBF and volume variations appear to be more strongly related to the degree of prematurity than to clinical complications.

Regarding MRI abnormalities, WM lesions were related to a significant decrease in overall and regional CBF. To explain this brain perfusion decrease, Tokumaru suggested that areas with WM lesions have fewer nerve fibers than healthy brain areas, and therefore have decreased myelination with lower metabolic needs (Tokumaru et al., 1999). However, in our study we showed that brain areas with lower perfusion are more extensive than areas with WM lesions, suggesting that WM lesions have an overall impact on brain myelination and maturation processes.

This study has several strengths. First, patient enrollment was prospective. Second, we used automated brain-region segmentation instead of the manual ROIs usually drawn for morphological, diffusion and ASL perfusion data analysis, ensuring faster processing of data and more reproducible results. Third, from the pipeline we developed, we extracted quantitative MRI parameters that enabled objective comparison between the preterm groups. Last, to the best of our knowledge this is the first study to combine morphological, diffusion and ASL brain perfusion analysis in preterm infants in order to ascertain the impact of the degree of prematurity and perinatal complications on brain maturation processes. We are aware that our study has certain limitations. First, the MRI examinations were performed on two different scanners and we used two types of morphological and diffusion sequences, which can cause bias. However, we observed that the sequence used had no effect on brain volumes and scalar diffusion parameters. Furthermore, volumetric measurements are relatively insensitive to the MRI scanner variations (Stonnington et al., 2008), and we adjusted the morphometric, diffusion and perfusion parameters based on a multiple linear regression model to consider these potential variations. Second, the MRI scan was performed a week later in extremely preterm infants as compared to very preterm infants. We showed that volumetric and diffusion parameters were strongly correlated with the age at MRI. Hence, we also included the age at MRI in our multiple linear regression model to adjust the MRI parameters. Last, we did not include moderate to late preterm infants (32–37 weeks’ gestation). At our center, we only perform brain MRI scans at term-equivalent age in extremely and very preterm infants because the risk of late neurocognitive disabilities is higher in these groups.

All preterm infants included in our study were enrolled in a larger study and will undergo a neurocognitive evaluation through the Ages and Stages Questionnaires (ASQ) at the age of two. It will be interesting to evaluate potential correlations between quantitative MRI parameters and mid-term neurocognitive development in these preterm infants.

## Conclusion

This study shows that MRI brain scans performed at term-equivalent age in preterm infants provide quantitative imaging parameters that differ with respect to the degree of prematurity. ASL perfusion and morphometric sequences with relative regional volume analysis are the most suitable MRI sequences for separating preterm infants according to their degree of prematurity. Extremely preterm infants have higher regional CBF, and frontal relative volumes than very preterm infants, but lower brainstem and insular relative volumes. Focal WM lesions have an overall impact on brain GM perfusion and are related to a significant decrease in overall and regional CBF. These results provide further knowledge of the preterm brain maturation process. Ongoing studies are still needed to determine whether or not these quantitative parameters also correlate with mid-term neurocognitive development.

## Data Availability Statement

The datasets presented in this article are not available in open access due to privacy restrictions. Indeed, the datasets consist in MRI scans acquired in preterm infants, and we did not obtain parental consent to make them publicly available. However, they consented for access to be granted to the journal reviewers to pseudonymized collected data so that they be examined and that the conclusions of the article be evaluated before its publication. Requests to access the datasets should be directed to MD, marine.dubois@chu-rennes.fr.

## Ethics Statement

The studies involving human participants were reviewed and approved by the Comité de Protection des Personnes Ouest IV CHU Nantes. Written informed consent to participate in this study was provided by the participants’ legal guardian/next of kin.

## Author Contributions

MD, J-CF, CB, and MP contributed to the conception and design of the study. PP included all the patients of the study. MD and MP acquired the MRI scans. MD, AL, IC, OC, and MP contributed to the data analysis and interpretation. MD, AL, and IC performed the statistical analysis. MD drafted the initial manuscript. All authors contributed to manuscript revision, read, and approved the submitted version.

## Conflict of Interest

The authors declare that the research was conducted in the absence of any commercial or financial relationships that could be construed as a potential conflict of interest.

## Footnotes

1 https://clinicaltrials.gov/ct2/show/NCT02863978

2 Open source software for medical image processing from the Empenn team, available at https://github.com/Inria-Visages/Anima-Public

3 Available at http://brain-development.org/brain-atlases/neonatal-brain-atlases/neonatal-brain-atlas-gousias/

4 Aavailable at https://github.com/Inria-Visages/Anima-Scripts-Public – RRID:SCR_017017

5 Available at http://developmentalimagingmcri.github.io/mantis

6 Open source software for medical image processing from the Empenn team, available at https://github.com/Inria-Visages/Anima-Public – RRID:SCR017017

## References

[B1] Alexander B. Kelly C. E. Adamson C. Beare R. Zannino D. Chen J. (2019). Changes in neonatal regional brain volume associated with preterm birth and perinatal factors. *NeuroImage* 185 654–663. 10.1016/j.neuroimage.2018.07.021 30016676

[B2] Alsop D. C. Detre J. A. Golay X. Günther M. Hendrikse J. Hernandez-Garcia L. (2015). Recommended implementation of arterial spin labeled perfusion MRI for clinical applications: a consensus of the ISMRM perfusion study group and the european consortium for ASL in dementia. *Magn. Reson. Med.* 73 102–116. 10.1002/mrm.25197 24715426 PMC4190138

[B3] Ashburner J. (2009). Computational anatomy with the SPM software. *Magnet. Reson. Imaging* 27 1163–1174. 10.1016/j.mri.2009.01.006 19249168

[B4] Beare R. J. Chen J. Kelly C. E. Alexopoulos D. Smyser C. D. Rogers C. E. (2016). Neonatal brain tissue classification with morphological adaptation and unified segmentation. *Front. Neuroinform* 10:12. 10.3389/fninf.2016.00012 PMC4809890 27065840

[B5] Beck S. Wojdyla D. Say L. Pilar Bertran A. Meraldi M. Harris Requejo J. (2010). The worldwide incidence of preterm birth: a systematic review of maternal mortality and morbidity. *Bull World Health Org.* 88 31–38. 10.2471/blt.08.062554 20428351 PMC2802437

[B6] Bouyssi-Kobar M. du Plessis A. J. McCarter R. Brossard-Racine M. Murnick J. Tinkleman L. (2016). Third trimester brain growth in preterm infants compared with in utero healthy fetuses. *Pediatrics* 138:e20161640. 10.1542/peds.2016-1640 27940782 PMC5079081

[B7] Bouyssi-Kobar M. Murnick J. Brossard-Racine M. Chang T. Mahdi E. Jacobs M. (2018). Altered cerebral perfusion in infants born preterm compared with infants born full term. *J. Pediatr.* 193 54–61.e2. 29212618 10.1016/j.jpeds.2017.09.083 PMC5794508

[B8] Buxton R. B. Frank L. R. Wong E. C. Siewert B. Warach S. Edelman R. R. (1998). A general kinetic model for quantitative perfusion imaging with arterial spin labeling. *Magnet. Reson. Med.* 40 383–396. 10.1002/mrm.1910400308 9727941

[B9] Chawanpaiboon S. Vogel J. P. Moller A.-B. Lumbiganon P. Petzold M. Hogan D. (2019). Global, regional, and national estimates of levels of preterm birth in 2014: a systematic review and modelling analysis. *Lancet Glob Health.* 7 e37–e46. 30389451 10.1016/S2214-109X(18)30451-0 PMC6293055

[B10] De Vis J. B. Hendrikse J. Groenendaal F. de Vries L. S. Kersbergen K. J. Benders M. J. N. L. (2014). Impact of neonate haematocrit variability on the longitudinal relaxation time of blood: implications for arterial spin labelling MRI. *NeuroImage Clin.* 4 517–525. 10.1016/j.nicl.2014.03.006 24818078 PMC3984444

[B11] De Vis J. B. Petersen E. T. de Vries L. S. Groenendaal F. Kersbergen K. J. Alderliesten T. (2013). Regional changes in brain perfusion during brain maturation measured non-invasively with Arterial Spin Labeling MRI in neonates. *Eur. J. Radiol.* 82 538–543. 10.1016/j.ejrad.2012.10.013 23199750

[B12] Gilmore J. H. Lin W. Prastawa M. W. Looney C. B. Vetsa Y. S. K. Knickmeyer R. C. (2007). Regional gray matter growth, sexual dimorphism, and cerebral asymmetry in the neonatal brain. *J. Neurosci.* 27 1255–1260. 10.1523/jneurosci.3339-06.2007 17287499 PMC2886661

[B13] Gousias I. S. Edwards A. D. Rutherford M. A. Counsell S. J. Hajnal J. V. Rueckert D. (2012). Magnetic resonance imaging of the newborn brain: manual segmentation of labelled atlases in term-born and preterm infants. *NeuroImage* 62 1499–1509. 10.1016/j.neuroimage.2012.05.083 22713673

[B14] Hales P. W. Kirkham F. J. Clark C. A. (2016). A general model to calculate the spin-lattice (T1) relaxation time of blood, accounting for haematocrit, oxygen saturation and magnetic field strength. *J. Cereb. Blood Flow. Metab.* 36 370–374. 10.1177/0271678x15605856 26661147 PMC4759664

[B15] Hédouin R. Commowick O. Bannier E. Scherrer B. Taquet M. Warfield S. K. (2017). Block-matching distortion correction of echo-planar images with opposite phase encoding directions. *IEEE Transact. Med. Imaging* 36 1106–1115. 10.1109/tmi.2016.2646920 28092527

[B16] Jopling J. Henry E. Wiedmeier S. E. Christensen R. D. (2009). Reference Ranges for hematocrit and blood hemoglobin concentration during the neonatal period: data from a multihospital health care system. *Pediatrics* 123 e333–e337. 19171584 10.1542/peds.2008-2654

[B17] Kim D.-Y. Park H.-K. Kim N.-S. Hwang S.-J. Lee H. J. (2016). Neonatal diffusion tensor brain imaging predicts later motor outcome in preterm neonates with white matter abnormalities. *Ital. J. Pediatr.* 42:104. 10.1186/s13052-016-0309-9 PMC5134238 27906083

[B18] Larroque B. Ancel P.-Y. Marret S. Marchand L. André M. Arnaud C. (2008). Neurodevelopmental disabilities and special care of 5-year-old children born before 33 weeks of gestation (the EPIPAGE study): a longitudinal cohort study. *Lancet* 371 813–820. 10.1016/s0140-6736(08)60380-3 18328928

[B19] Le Bihan D. (2013). Apparent diffusion coefficient and beyond: what diffusion MR imaging can tell us about tissue structure. *Radiology* 268 318–322. 10.1148/radiol.13130420 23882093

[B20] Lebel C. Treit S. Beaulieu C. (2019). A review of diffusion MRI of typical white matter development from early childhood to young adulthood. *NMR Biomed.* 32:e3778. 10.1002/nbm.3778 28886240

[B21] Legouhy A. Commowick O. Rousseau F. Barillot C. (2019). *Unbiased Longitudinal Brain Atlas Creation Using Robust Linear Registration and Log-Euclidean Framework for Diffeomorphisms.* Available online at: https://www.hal.inserm.fr/inserm-02099958 (accessed June 11, 2019).

[B22] Lemola S. Oser N. Urfer-Maurer N. Brand S. Holsboer-Trachsler E. Bechtel N. (2017). Effects of gestational age on brain volume and cognitive functions in generally healthy very preterm born children during school-age: a voxel-based morphometry study. *PLoS One* 12:e0183519. 10.1371/journal.pone.0183519 28850616 PMC5574554

[B23] Luh W.-M. Wong E. C. Bandettini P. A. Hyde J. S. (1999). QUIPSS II with thin-slice TI1 periodic saturation: a method for improving accuracy of quantitative perfusion imaging using pulsed arterial spin labeling. *Magnet. Reson. Med.* 41 1246–1254. 10.1002/(sici)1522-2594(199906)41:6<1246::aid-mrm22>3.0.co;2-n 10371458

[B24] Padilla N. Alexandrou G. Blennow M. Lagercrantz H. Ådén U. (2015). Brain growth gains and losses in extremely preterm infants at term. *Cerebral Cortex.* 25 1897–1905. 10.1093/cercor/bht431 24488941

[B25] Papile L.-A. Burstein J. Burstein R. Koffler H. (1978). Incidence and evolution of subependymal and intraventricular hemorrhage: a study of infants with birth weights less than 1,500 gm. *J. Pediatr.* 92 529–534. 10.1016/s0022-3476(78)80282-0 305471

[B26] Paquette N. Shi J. Wang Y. Lao Y. Ceschin R. Nelson M. D. (2017). Ventricular shape and relative position abnormalities in preterm neonates. *Neuroimage Clin.* 15 483–493. 10.1016/j.nicl.2017.05.025 28649491 PMC5470570

[B27] Pecheva D. Kelly C. Kimpton J. Bonthrone A. Batalle D. Zhang H. (2018). Recent advances in diffusion neuroimaging: applications in the developing preterm brain. *F1000Res* 7:F1000FacultyRev-1326. 10.12688/f1000research.15073.1 PMC6107996 30210783

[B28] Proisy M. Corouge I. Leghouy A. Nicolas A. Charon V. Mazille N. (2019). Changes in brain perfusion in successive arterial spin labeling MRI scans in neonates with hypoxic-ischemic encephalopathy. *Neuroimage Clin.* 24:101939. 10.1016/j.nicl.2019.101939 31362150 PMC6664197

[B29] Proisy M. Mitra S. Uria-Avellana C. Sokolska M. Robertson N. J. Jeune F. L. (2016). Brain perfusion imaging in neonates: an overview. *Am. J. Neuroradiol.* 37 1766–1773. 10.3174/ajnr.a4778 27079367 PMC7960464

[B30] Serag A. Aljabar P. Ball G. Counsell S. J. Boardman J. P. Rutherford M. A. (2012). Construction of a consistent high-definition spatio-temporal atlas of the developing brain using adaptive kernel regression. *NeuroImage* 59 2255–2265. 10.1016/j.neuroimage.2011.09.062 21985910

[B31] Shim S.-Y. Jeong H.-J. Son D. W. Jeong J. S. Oh S. H. Park S.-Y. (2012). Altered microstructure of white matter except the corpus callosum is independent of prematurity. *NEO* 102 309–315. 10.1159/000341867 22986463

[B32] Smyser T. A. Smyser C. D. Rogers C. E. Gillespie S. K. Inder T. E. Neil J. J. (2016). Cortical gray and adjacent white matter demonstrate synchronous maturation in very preterm infants. *Cereb. Cortex.* 26 3370–3378. 10.1093/cercor/bhv164 26209848 PMC4961016

[B33] Stonnington C. M. Tan G. Klöppel S. Chu C. Draganski B. Jack C. R. (2008). Interpreting scan data acquired from multiple scanners: a study with Alzheimer’s disease. *Neuroimage* 39 1180–1185. 10.1016/j.neuroimage.2007.09.066 18032068 PMC2225446

[B34] Tokumaru A. M. Barkovich A. J. O’uchi T. Matsuo T. Kusano S. (1999). The evolution of cerebral blood flow in the developing brain: evaluation with iodine-123 iodoamphetamine SPECT and correlation with MR imaging. *AJNR Am. J. Neuroradiol.* 20 845–852. 10369355 PMC7056162

[B35] Tolsa C. B. Zimine S. Warfield S. K. Freschi M. Rossignol A. S. Lazeyras F. (2004). Early alteration of structural and functional brain development in premature infants born with intrauterine growth restriction. *Pediatr. Res.* 56 132–138. 10.1203/01.pdr.0000128983.54614.7e 15128927

[B36] Tortora D. Mattei P. A. Navarra R. Panara V. Salomone R. Rossi A. (2017). Prematurity and brain perfusion: arterial spin labeling MRI. *Neuroimage Clin.* 15 401–407. 10.1016/j.nicl.2017.05.023 28603687 PMC5454138

[B37] Wintermark M. Sesay M. Barbier E. Borbély K. Dillon W. P. Eastwood J. D. (2005). Comparative overview of brain perfusion imaging techniques. *Stroke* 36 e83–e99. 16100027 10.1161/01.STR.0000177884.72657.8b

[B38] Wu D. Chang L. Akazawa K. Oishi K. Skranes J. Ernst T. (2017). Change-point analysis data of neonatal diffusion tensor MRI in preterm and term-born infants. *Data Brief.* 12 453–458. 10.1016/j.dib.2017.04.020 28516143 PMC5426014

